# Supplementary material for: Concurrent Infection of the Human Brain with Multiple Borrelia Species
Source: Int J Mol Sci. 2023 Nov 29;24(23):16906. doi: 10.3390/ijms242316906 (PMC10707132; doi:10.3390/ijms242316906)
Supplement: Supplementary file 1 [file ijms-24-16906-s001.zip › ijms-2682924-supplementary.pdf]

Supplementary Material and Methods

Induction of formation of atypical morphologies of Borrelia by antibiotics

Spirochetes of *B. burgdorferi* s.s. strain NE-5264 were grown in modified Kelly-Pettenkofer medium (MKP) (122). The cultures were incubated at 33°C until cell density reached at least 10^6 spirochetes per milliliter. The absence of contamination and the viability of spirochetes was verified by microscopy. The spirochete concentration was determined using a Petroff-Hausser counting chamber. Five sterile DNase-free Eppendorf tubes with spirochete cultures were treated with antibiotics doxycycline or amoxicillin (Sigma-Aldrich, Burlington, MA, USA) regularly used in the treatment of LD. Each antibiotic was applied at two concentrations: 50 μg/ml and 100 μg/ml. An untreated culture was used as a positive control. After 14 days of antibiotic treatment, the spirochetes (7.5×10^7) were washed in 0.1 M HEPES, pelleted by centrifugation (820 × g, 10 min), fixed in 4% formaldehyde with 0.1% glutaraldehyde in 0.1M HEPES for 1h at RT and immediately transferred to freshly prepared 2% agar for processing of paraplast sections.

Infection of laboratory mice (control)

Susceptible to *Borrelia* mice C3H/HeN genotype were used as laboratory animal model for control experiments. Six weeks old female mice (Charles River, Koln, Germany) were infected by simultaneous subcutaneous and intraperitoneal injections of 10^4 replicating spirochetes in 100 μl of MKP medium per mouse.

Immunohistochemical detection of cultured Borrelia

Immunodetection of *Borrelia* on paraplast sections of cultured spirochetes was performed using the same protocol as for human brain tissue. A specifically bound primary antibody was detected by incubation with the goat anti-rabbit IgG conjugated Alexa Fluor 488 secondary antibody (Life Technologies, Carlsbad, CA, USA), diluted 1: 500 in the blocking solution, for 90 minutes at RT in dark.

DNA extraction

For total DNA purification from all collected samples, the DNeasy Blood and Tissue kit (Qiagen, Hilden, Germany) were used. To increase DNA yield and so the possibility of capture of spirochete DNA in the sample, the entire frozen tissue was weighted, homogenized in liquid nitrogen and then subsequently processed according to the manufacturer's protocol.

Sequencing

All amplicons of the expected sizes were excised from agarose gels, purified using QIAquick PCR Purification Kit (QIAGEN, Hilden, Germany) and sequenced in both directions using the same primers used for amplification. Sequence analysis was performed commercially by SEQme s.r.o. (Dobris, Czech Republic) and the sequences were compared to those available in the NCBI GenBan database using Basic Local Alignment Tool (BLASTn) analysis.

Cultivation of Borrelia from CSF samples

Five hundred microliters of CSF were transferred to a 5 ml of Barbour-Stoner-Kelly culture medium (BioConcept, Allschwil, Switzerland) supplemented with 6% rabbit serum (Merck, Rahway, NJ, USA) and antibiotics phosphomycin, polymyxin and rifampicin (100x concentrated solution, HiMedia, India diluted 1:100). Seeded cultures were kept at +33°C for two months with regular checks by dark-field microscopy, starting from day 10 after culture initiation.

Transmission electron microscopy

Spirochetes were fixed in 2.5% glutaraldehyde in 0.1M PBS for 1h at RT. Cells were washed three times in 0.1 M PBS with 4% glucose, embedded into 2% of agar, and postfixed in 2% OsO_4 for 1h at RT. After washing, samples were dehydrated stepwise using a graded acetone series (30-50-70-80-90-95%, v/v) for 15 min at each step and transferred to absolute acetone for 15 min. Samples were infiltrated in 2:1, 1:1, and 1:2 mixtures from acetone/stock resin solutions (1h/each step) and finally in two changes of Poly/Bed 812 resin (Polysciences Inc., Warrington, PA, USA) before embedding and polymerization. Ultrathin sections were stained in saturated ethanolic uranyl acetate and lead citrate before imaging in JEM-1400 Flash TEM (JEOL Ltd.).

Reference

1. Preac-Mursic V, Wilske B, Schierz G. 1986. European *Borrelia burgdorferi* isolated from humans and ticks. Culture conditions and antibiotic susceptibility. Zentralbl Bakteriol Mikrobiol Hyg 263:112–118

Supplementary Table S1

Target	Primer	GenBank accession number	5' – 3' nucleotide sequence	Position 5' (nt)*	Reference
ospC	F1 Ext	U01894	ATGAAAAAGAATACATTAAGTGC	306	Bunikis et al. 2004
	R1 Ext		ATTAATCTTATAATATTGATTTTAATTAAGG	933	
	F2 Int		TATTAATGACTTTATTTTTATTTATATCT	331	
	R2 Int		TTGATTTTAATTAAGGTTTTTTTGG	924	
flagellin	F1 Ext	X15661	AARGAATTGGCAGTTCAATC	271	Clark et al. (2005)
	R1 Ext		GCATTTTCWATTTTAGCAAGTGATG	767	
	F2 Int		ACATATTCAGATGCAGACAGAGGTTCTA	301	
	R2 Int		GAAGGTGCTGTAGCAGGTGCTGGCTGT	663	
clpA	F1 Ext	BB0369	GATAGATTTCTTCCAGACAAAG	1240	Margos et al. 2008
	R1 Ext		TTCATCTATTAAAAGCTTTCCC	2214	
	F2 Int		GACAAAGCTTTTGATATTTTAG	1255	
	R2 Int		CAAAAAAAACATCAAATTTTCTATCTC	2104	
clpX	F1 Ext	BB0612	GCTGCAGAGATGAATGTGCC	391	Margos et al. 2008
	R1 Ext		GATTGATTTCATATAACTCTTTTG	1273	
	F2 Int		AATGTGCCATTTGCAATAGC	403	
	R2 Int		TTAAGAAGACCCTCTAAAATAG	1124	
pyrG	F1 Ext	BB0575	GATTGCAAGTTCTGAGAATA	391	Margos et al. 2008
	R1 Ext		CAAACATTACGAGCAAATTC	1190	
	F2 Int		GATATGGAAAATATTTTATTTATTG	448	
	R2 Int		AAACCAAGACAAATTCCAAG	1154	
uvrA	F1 Ext	BB0837	GAAATTTTAAAGGAAATTAAAAGTAG	1408	Margos et al. 2008
	R1 Ext		CAAGGAACAAAAACATCTGG	2318	
	F2 Int		GCTTAAATTTTTAATTGATGTTGG	1434	
	R2 Int		CCTATTGGTTTTTGATTTATTTG	2111	
pepX	F1 Ext	BB0627	ACAGAGACTTAAGCTTAGCAG	362	Margos et al. 2008
	R1 Ext		GTTCCAATGTCAATAGTTTC	1172	
	F2 Int		TTATTCCAAACCTTGCAATCC	449	
	R2 Int		TGTGCCTGAAGGAACATTTG	1115	
recG	F1 Ext	BB0581	CCCTTGTTGCCTTGCTTTC	890	Margos et al. 2008
	R1 Ext		GAAAGTCCAAAACGCTCAG	1694	
	F2 Int		CTTTAATTGAAGCTGGATATC	917	
	R2 Int		CAAGTTGCATTTGGACAATC	1658	
rplB	F1 Ext	BB0481	TGGGTATTAAGACTTATAAGC	2	Margos et al, 2008
	R1 Ext		GCTGTCCCCAAGGAGACA	760	
	F2 Int		CGCTATAAGACGACTTTATC	40	
	R2 Int		GCTGTCCCCAAGGAGACA	760	
nifS	F1 Ext	BB0084	ATGGATTTCAAACAAATAAAAAG	1	Margos et al. 2008
	R1 Ext		GATATTATTGAATTTCTTTTAAG	719	
	F2 Int		ATGGATTTCAAACAAATAAAAAG	1	
	R2 Int		GTTGGAGCAAGCATTTTATG	680	

Supplementary Figure S1

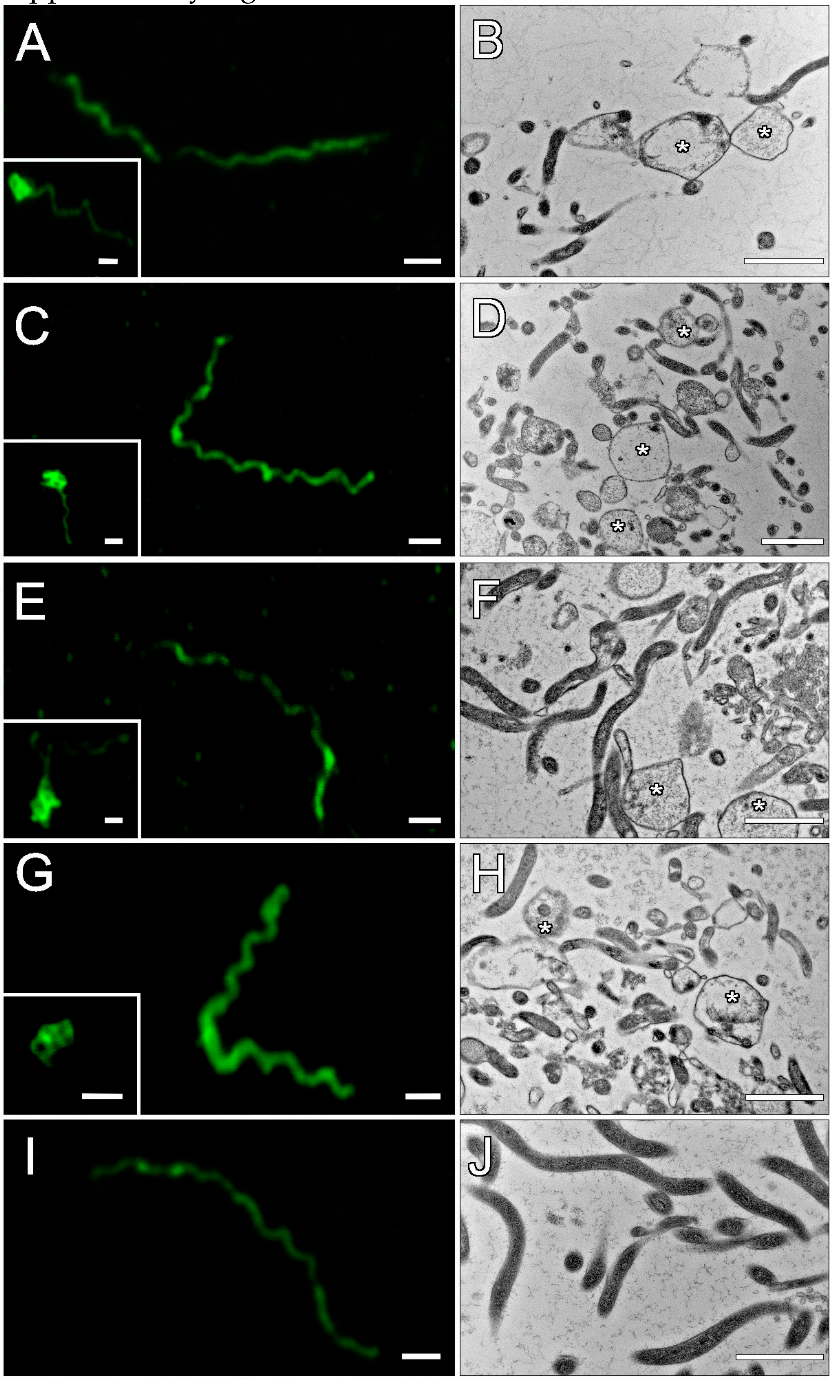

Supplementary Figure 1. Spiral and atypical Borrelia forms detected after antibiotics treatment. Immunofluorescence and TEM images of B. burgdorferi s.s. cultured with amoxycillin (A-D) and doxycycline (E-H) antibiotics at concentrations of 50 µg/ml (A, B, E, F) and 100 µg/ml (C, D, G, H). Both spiral and atypical (asterisk) morphological

forms were observed in antibiotics-treated cultures in contrast to control (I, J). Scale: 1 μm.
